# Adiponectin receptor 1 conserves docosahexaenoic acid and promotes photoreceptor cell survival

**DOI:** 10.1038/ncomms7228

**Published:** 2015-03-04

**Authors:** Dennis S. Rice, Jorgelina M. Calandria, William C. Gordon, Bokkyoo Jun, Yongdong Zhou, Claire M. Gelfman, Songhua Li, Minghao Jin, Eric J. Knott, Bo Chang, Alex Abuin, Tawfik Issa, David Potter, Kenneth A. Platt, Nicolas G. Bazan

**Affiliations:** 1 Lexicon Pharmaceuticals, 8800 Technology Forest Place, The Woodlands, Texas 77381, USA; 2 Neuroscience Center of Excellence, School of Medicine, Louisiana State University Health Sciences Center, 2020 Gravier Street, New Orleans, Louisiana 70112, USA; 3 The Jackson Laboratory, 600 Main Street, Bar Harbor, Maine 04609, USA

## Abstract

The identification of pathways necessary for photoreceptor and retinal pigment epithelium (RPE) function is critical to uncover therapies for blindness. Here we report the discovery of adiponectin receptor 1 (AdipoR1) as a regulator of these cells’ functions. Docosahexaenoic acid (DHA) is avidly retained in photoreceptors, while mechanisms controlling DHA uptake and retention are unknown. Thus, we demonstrate that AdipoR1 ablation results in DHA reduction. *In situ* hybridization reveals photoreceptor and RPE cell AdipoR1 expression, blunted in AdipoR1^−/−^ mice. We also find decreased photoreceptor-specific phosphatidylcholine containing very long-chain polyunsaturated fatty acids and severely attenuated electroretinograms. These changes precede progressive photoreceptor degeneration in AdipoR1^−/−^ mice. RPE-rich eyecup cultures from AdipoR1^−/−^ reveal impaired DHA uptake. AdipoR1 overexpression in RPE cells enhances DHA uptake, whereas AdipoR1 silencing has the opposite effect. These results establish AdipoR1 as a regulatory switch of DHA uptake, retention, conservation and elongation in photoreceptors and RPE, thus preserving photoreceptor cell integrity.

## Abstract (web summary)

Docosahexaenoic acid is a major and important retinal fatty acid that is recruited and retained in the photoreceptor membrane via an unknown mechanism. Here, Rice *et al*. show that adiponectin receptor 1 is a key molecular switch for docosahexaenoic acid membrane homeostasis and photoreceptor cell function.

## Graphical abstract

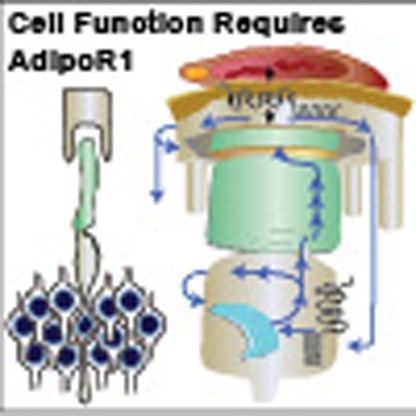

Docosahexaenoic acid (DHA, 22:6ω3), an omega-3 essential fatty acid (FA), is avidly retained and concentrated in the central nervous system (CNS), attaining its highest concentration in photoreceptor cells (PRCs)^1^. After DHA uptake by RPE cells from the choriocapillaris, DHA, through the interphotoreceptor matrix, reaches photoreceptor inner segments, where it is taken up and acylated to phospholipids for photoreceptor outer segment (OS) membrane biogenesis^1^. As a consequence of the uptake and specificity of the molecular systems engaged, DHA ends up accounting for over 50% of OS phospholipid fatty acyl chains. Moreover, DHA-containing phospholipids in the retina and other cells are reservoirs for potent bioactive mediators, the docosanoids^2^,^3^. The docosanoid, neuroprotectin D1 (NPD1), is a stress-injury response mediator made on-demand when homeostatic disruptions such as protein misfolding, Aβ peptide challenge and/or uncompensated oxidative stress emerge^1^, prompting responses to counteract neuroinflammation consequences including neurodegeneration. While there is growing evidence of the significance of DHA for photoreceptor function and in retinal degenerative diseases, we have a void in our understanding of the molecular events governing DHA-selective retention/conservation, both in the RPE and in PRCs. We have discovered that the adiponectin receptor 1 (AdipoR1) regulates DHA retention and is necessary for PRC function. Upon AdipoR1 ablation, DHA uptake is impaired, photoreceptor-specific very long-chain polyunsaturated FA (VLC-PUFA)-containing phosphatidylcholine (PC) molecular species are reduced, and photoreceptor function and survival are compromised.

AdipoR1 has been extensively studied as a receptor for the hormone adiponectin, which promotes insulin sensitivity, has anti-inflammatory properties and is a pro-cell survival factor^4^,^5^. Adiponectin binding to AdipoR1 is associated with stimulation of adenosine monophosphate-activated kinase activity in some cell types, and the downstream signalling is being explored^6^,^7^. Moreover, adiponectin promotes AdipoR1-dependent ceramidase activity to regulate ceramide levels^8^. Although AdipoR1 is a seven-transmembrane domain protein, it does not signal through G-protein-coupled mechanisms, and, unlike most of this type of receptor, the N terminus is intracellular and the C terminus is extracellular^5^. Recently, adiponectin and its receptors were reported in type 1 diabetes mellitus in human and mouse retinas^9^.

Genetic knockouts (KOs) of AdipoR1 and AdipoR2 have demonstrated adiponectin-induced signalling in skeletal muscle and liver^5^,^10^. However, AdipoR1 mRNA is expressed in many tissues, and the CNS exhibits abundant AdipoR1 expression. High levels of AdipoR1 are observed in the retinal pigment epithelium (RPE) and neural retina as illustrated in the gene expression portal, BioGPS^11^,^12^. Recently, a SNP in the human AdipoR1 locus was associated with age-related macular degeneration (AMD) in a Finnish population^13^, but functional and mechanistic data for AdipoR1 in photoreceptors are lacking.

Here we discovered a novel function of the integral membrane protein AdipoR1 that is necessary for PRC integrity and survival. We found that this protein mediates DHA retention in RPE and PRCs. We created two independent lines of AdipoR1 KO mice by retroviral gene trapping and homologous recombination, which, in turn, foster photoreceptor degeneration. Using *in situ* hybridization, we show that AdipoR1 occurs in photoreceptor/RPE cells, whereas no specific signal appears in mice lacking AdipoR1. Thus, we demonstrate that ablation of this receptor shows: (a) progressive PRC degeneration (3–33 weeks of age by optical coherence tomography (OCT) and histology), attenuated electroretinograms (ERGs) and a severely and early impaired retinol visual cycle; (b) a flecked retina resembling human *fundus albipunctatus* with intact vasculature (12–16 weeks old); (c) anti-F4/80-positive cells (activated macrophages) beneath the RPE, UV autofluorescence in RPE and macrophages, and undigested OS debris in the RPE (by EM); (d) terminal deoxynucleotidyl transferase dUTP nick end labeling (TUNEL)-positive cells in the outer nuclear layer (ONL); (e) specific reduction of retinal DHA, since arachidonic acid (esterified and free) and systemic DHA were unchanged; (f) decreased DHA uptake in *ex vivo* explants of eyecups/RPE incubated with deuterium-labelled DHA; (g) overexpression or silencing of AdipoR1 in human RPE cells, leading to enhanced or decreased DHA uptake, respectively; (h) absence of PRC-specific VLC-PUFAs, along with unchanged ELongase Of the Very Long-chain fatty acids-4 (ELOVL4) abundance; and (i) concomitantly, a set of minor VLC-PUFAs, not containing DHA, are formed in the AdipoR1^−/−^ retinas, perhaps as a compensatory response from damaged photoreceptors or from Müller or monocytes/microglial cells to pathology. Furthermore, we show that the adiponectin KO is devoid of a retinal degeneration phenotype. We thereby demonstrate that AdipoR1 is a novel molecular switch, independent of its cognate ligand, adiponectin, that selectively and specifically controls the DHA lipidome in RPE and PRCs. Moreover, this switch modulates DHA retention and conservation, and is required for photoreceptor-specific elongation to VLC-PUFAs. We hypothesize that since the photoreceptor DHA lipidome comprises endogenous cell survival responses, mimicking them to counteract early stages of retinal degenerative diseases will lead to a therapeutic paradigm shift.

## Results

### Retinal degeneration is triggered by AdipoR1 ablation

**Figure 1:**
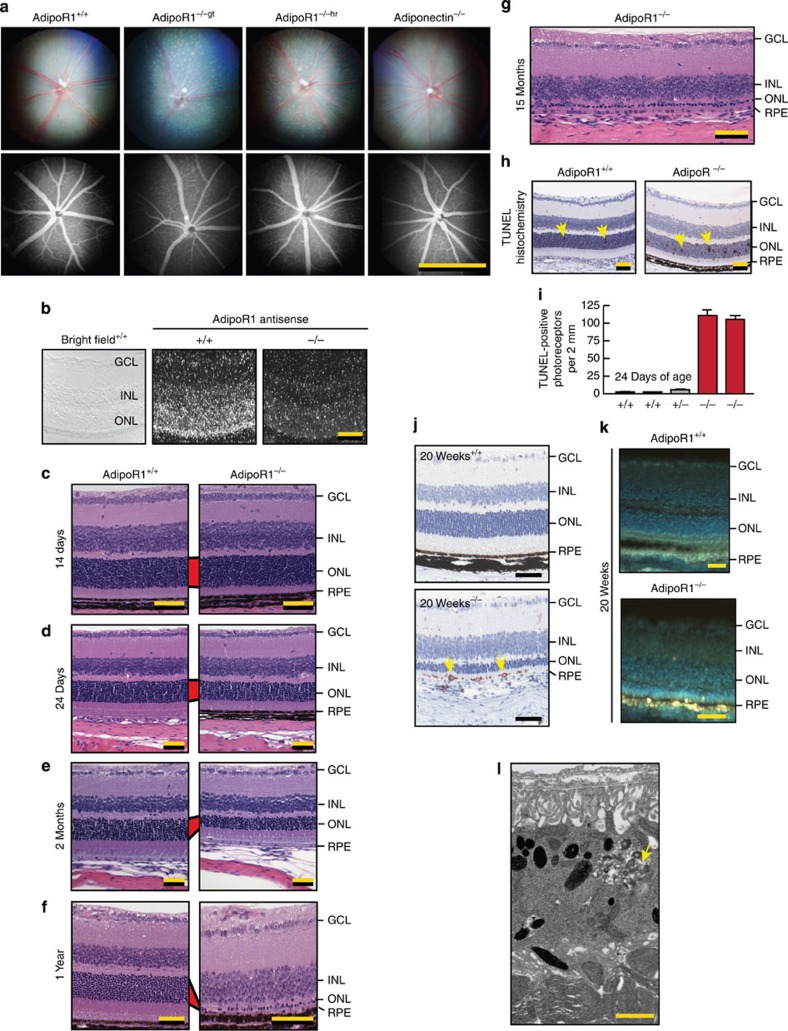
Ablation of AdipoR1 results in PRC degeneration. (**a**) Fundus images of AdipoR1^+/+^ and two independent lines of AdiopR1 KO mice, one created using gene trapping (AdipoR1^−/−gt^) and the other using homologous recombination (AdipoR1^−/−hr^). Mice are between 14 and 16 weeks of age. Light spots, resulting from subretinal macrophages, appear as a ‘flecked retina’ in the fundus images captured from mice deficient in AdipoR1, regardless of the gene-targeting strategy. The Adiponectin^−/−^ fundus (AdipoQ^−/−^) was normal and showed no macrophage infiltration or retinal degeneration. Angiograms demonstrate intact vasculature. (**b**) *In situ* hybridization of control (^+/+^) and AdipoR1 KO (^−/−^) (AdipoR1^gt^) mouse eyes revealed intense expression in the outer nuclear layer (ONL). Weaker signals were observed in the inner nuclear layer (INL) and ganglion cell layer (GCL). The brightfield image was obtained from the same AdipoR1^+/+^ section shown in darkfield in the middle panel. No specific signal was observed in AdipoR1^−/−^ retinas hybridized to the same antisense probe. (**c**–**g**) Progressive loss of PRCs occurred in AdipoR1^−/−^ retinas. Similar anatomy is observed when comparing AdipoR1^+/+^ with AdipoR1^−/−^ retinas at 14 days of age (**c**). Slight ONL thinning occurred at 24 days (**d**) and by 2 months, 50% of AdipoR1^−/−^ photoreceptors have degenerated (**e**). A single row of photoreceptors remained in AdipoR1^−/−^ mice at 1 year (**f**), or older (15 months, **g**), while AdipoR1^+/+^ retinas retained normal ONL thicknesses of 11–12 nuclei. No inner retina degeneration occurred. (Red bars connecting the ONLs of AdipoR1^+/+^ and ^−/−^ retinas highlight progressive photoreceptor loss. (**h**,**i**) Many photoreceptor nuclei have fragmented DNA (TUNEL assay), indicating onset of apoptosis (arrows) within the 24-day-old AdipoR1^−/−^ retinas. (**j**) Immunolabelled anti-F4/80-positive cells (brown label, arrows) mark activated macrophages at the PR-RPE interface in 20-week-old AdipoR1^−/−^ mice, suggesting the onset of an inflammatory event. (**k**) Unstained sections of 20-week-old retinas under ultraviolet illumination revealed RPE and macrophages intensely autofluorescent (yellow regions), indicating accumulation of undigested outer segments. (**l**) Electron micrograph of retinal pigment epithelium (RPE) from a 5-month AdipoR1^−/−^ retina containing cellular debris undergoing digestion (arrow). Magnification bars: (**a**), 1 mm; (**b**–**k**) 50 μm; (**l**) 1 μm.

During a phenotype screen in KO mice, fundoscopy of 12- to 16-week-old AdipoR1 KO mice revealed a flecked retinal syndrome resembling human *fundus albipunctatus*, which is associated with a recessive mutation in 11-*cis*-retin*ol* dehydrogenase (RDH5), the enzyme that converts 11-*cis*-retin*ol* into 11-*cis*-retin*al* in the retinoid cycle^14^,^15^. This phenotype was observed in two independent lines of AdipoR1 KOs (Fig. 1a). Angiography revealed intact retinal vasculature (Fig. 1a). Thus, AdipoR1 may be important in the outer retina, where the visual cycle plays a key role in light perception. Mice lacking adiponectin, the cognate ligand of AdipoR1, exhibited no discernible retinal phenotype. An AdipoR1-specific riboprobe was hybridized to cryosections of AdipoR1^+/+^ and AdipoR1^−/−^ retinas (Fig. 1b), and high abundance of *AdipoR1* was observed in AdipoR1^+/+^ photoreceptors, whereas no specific hybridization signal was detected in AdipoR1^−/−^ tissue, confirming the genetic disruption of *AdipoR1*.

**Figure 2:**
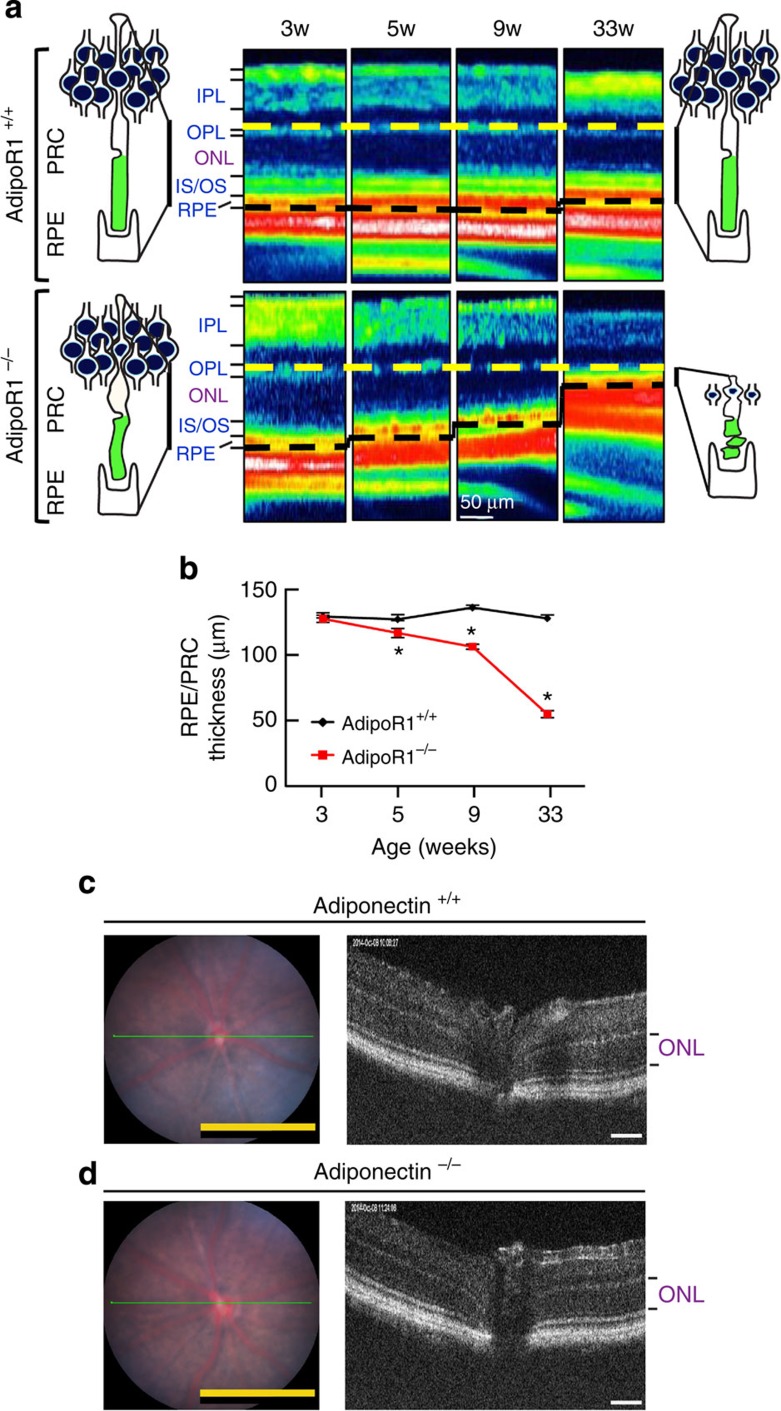
Progressive loss of photoreceptors in the AdipoR1^−/−^ mouse. Thickness of the photoreceptor layer (ONL), obtained by OCT, is comparable in 3-week-old AdipoR1^+/+^ and AdipoR1−/−. Loss of PRCs is apparent at 5 weeks of age, and then slowly declines, with 50% loss at ~13 weeks of age. (**a**) Images of AdipoR1^+/+^ mouse retinas showing an unchanging outer retina (RPE/PR, photoreceptor/retinal pigment epithelial layers combined) thickness (region between the dashed lines) from 3 weeks through 33 weeks of age (top row). Images of AdipoR1^−/−^ mouse retinas (bottom row) demonstrating a constant decline in RPE/PRC thickness from 5–33 weeks of age. Retinal layers are indicated at the left. (**b**) Graphic representation of retinal degeneration (*n*=5 per geneotype). s.e.m. is indicated by upward bars (* denotes significant differences between the genotypes at specific time points; *P*<0.05, determined by *t*-test). (**c**) Representative fundus and OCT imaging of an Adiponectin^+/+^ mouse retina. (**d**) Representative fundus and OCT imaging of an Adiponectin^−/−^ mouse retina. Panels **c**,**d** illustrate intact ONLs in 5-week-old mice, signifying no photoreceptor loss as a result of Adiponectin knockout. In **c**,**d**, three animals each were observed; magnification bar in fundus images at left is 1 mm, and 50 μm for OCT images at right. IPL, inner plexiform layer; OPL, outer plexiform layer; ONL, outer nuclear layer; RPE, retinal pigment epithelial cell layer; OR, outer retina.

OCT and histology revealed progressive retinal degeneration in AdipoR1^−/−^ mice between 3 and 33 weeks of age. No anatomical differences were discernible at 3 weeks; however, the photoreceptor layer thickness in AdipoR1^−/−^ mice declined thereafter, and by 33 weeks only a thin layer of photoreceptors remained (Fig. 2a,b). Fundus and corresponding OCT images from Adiponectin^+/+^ and ^−/−^ retinas (AdipoQ) demonstrated no photoreceptor loss in the KO retinas (Fig. 2c,d). Histology of 14-day-old AdipoR1^+/+^ and AdipoR1^−/−^ mice revealed three intact nuclear layers with no discernible ONL thinning in AdipoR1^−/−^ mice (Fig. 1c). However, following a subtle decrease in ONL thickness at 3 weeks, thinning progressed to one row of nuclei at 12–15 months (Fig. 1c–g). Many TUNEL-positive cells appeared in the ONL of AdipoR1-deficient mice on day 24 (Fig. 1h,i). Fundoscopy flecks were determined to be F4/80-positive cells located at the photoreceptor–RPE interface (Fig. 1j). Unstained cryosections of AdipoR1^−/−^ retinas (20 weeks) revealed intensely autofluorescent RPE and macrophages under ultraviolet illumination, indicating undigested photoreceptor–OS (Fig. 1k), and electron microscopy revealed accumulated membrane debris (Fig. 1l). Thus, AdipoR1 is required for photoreceptor survival.

### Photoreceptor physiology is compromised in AdipoR1^−/−^ mice

**Figure 3:**
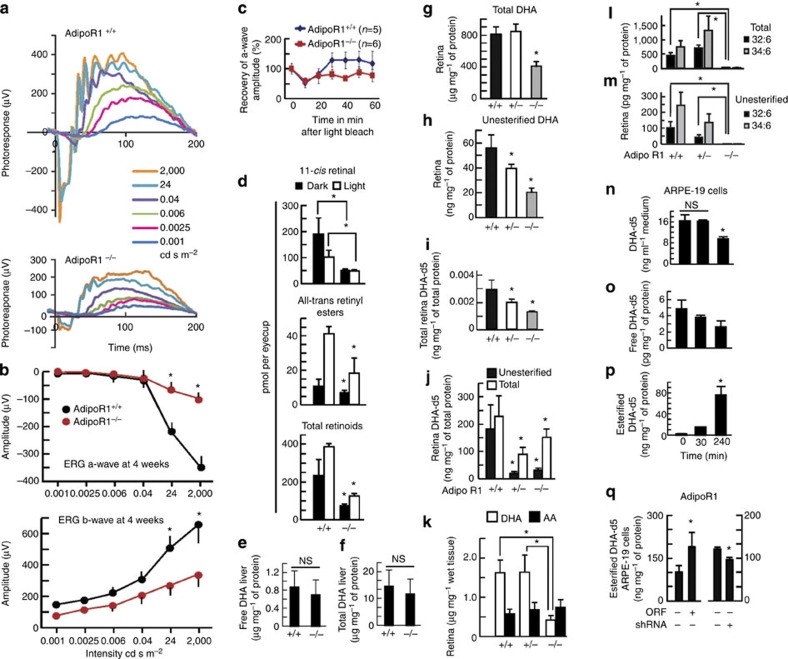
ERGs and visual cycle attenuated in AdipoR1^−/−^ mice. ERGs (3–4-week-old) before photoreceptor loss. (**a**) AdipoR1^−/−^ had reduced ERGs. (**b**) AdipoR1^−/−^ had attenuated a-/b-waves (**a**,**b**, *n*=4, AdipoR1^+/+^, *n*=6, AdipoR1^−/−^). (**c**) Impaired AdipoR1^−/−^ recovery following 5-min bleaching light. Differences in AdipoR1^+/+^ and ^−/−^ a-waves demonstrated impaired recovery trend (*n*=5, AdipoR1^+/+^, *n*=6, AdipoR1^−/−^ mice: 4–6-week old). (**d**) Impaired retinoid visual cycle (3–4-month old). (Dark-adapted mice 2,000 Lux, 10 min). 11-*cis*-retin*al*, all-*trans*-retinyl esters and total retinoids were diminished in AdipoR1^−/−^ in light and darkness (*n*=4: both genotypes). Liver DHA is not altered in AdipoR1^−/−^ mice. (**e**,**f**) Total and unesterified DHA showed no differences between 12-day AdipoR1^+/+^ and ^−/−^ (*n*=6 each). DHA uptake is reduced in AdipoR1^−/−^ mice. (**g**,**h**) Total and unesterified DHA (20-day-old AdipoR1^+/+^, *n*=14, ^+/−^ *n*=31 and ^−/−^ *n*=7. Total DHA declined within AdipoR1^−/−^; unesterified DHA declined in AdipoR1^+/−^ (30%) and AdipoR1^−/−^ (60%). AdipoR1 deficiency reduced DHA uptake. (**i**) AdipoR1^+/+^ (*n*=6), ^+/−^ (*n*=20), and ^−/−^ (*n*=5) were injected ip with DHA-d5 (14 days), and retinas harvested (20 days). DHA uptake declined in AdipoR1^−/−^; intermediate DHA levels occurred in AdipoR1^+/−^, indicating a single allele cannot control DHA levels. (**j**) Eye cup cultures (*n*=5, all three genotypes, 20-days-old) incubated (4 h) with DHA-d5. AdipoR1 loss resulted in reduction of total DHA in AdipoR1^+/−^ (60%) and ^−/−^ (30%) retinas. AdipoR1 selectively regulates DHA retinal uptake. (**k**) Total arachidonic acid (AA; 28-day-old mice) was similar in AdipoR1^+/+^ and ^−/−^, while AdipoR1^−/−^ DHA was decreased (75%). PC-associated VLC-PUFAs were reduced in AdipoR1^−/−^, with almost complete loss of total and unesterified 32:6 and 34:6 (**l**,**m**). (AdipoR1^+/+^, *n*=17; AdipoR1^+/−^, *n*=17; AdipoR1^−/−^, *n*=11). ARPE-19 cells incubated with DHA-d5 (100 nM) showed (**n**) media DHA-d5 decreased (50%), indicating DHA uptake, unesterified DHA declined (**o**) and DHA esterification increased (**p**), indicating DHA phospholipid incorporation. ARPE-19 cells incubated with DHA-d5 (240 min) and AdipoR1 overexpression enhanced DHA uptake and esterification; silencing decreased uptake and incorporation (**q**). (See AdipoR1 protein levels: Supplementary Fig. 1.) (**n**–**q**, statistical bars are s.e.m. of three for each condition. Experiments conducted three times. In all analyses, error bars represent s.e.m., and *P<0.05: *t*-test. NS=nonsignificant *P* value.)

ERGs were recorded from 3- to 4-week AdipoR1^−/−^ mice (24 days when ONL thicknesses were comparable to AdipoR1^+/+^ mice) at light intensities ranging from 24 to 2,000 cd s m^−2^ that initiate a mixed rod and cone photoreceptor response. Mice lacking AdipoR1 showed a dramatic 75% attenuation in a-wave amplitudes (photoreceptor response) compared with cohort, age-matched controls, while b-wave amplitudes (inner retina response) were depressed 50% (Fig. 3a,b). Mice were exposed to a brief bleaching light, followed by recording of a-wave amplitudes at increasing times to determine dark recovery. Figure 3c displays a trend of reduced recovery supported by a remarkable drop in retinoids in RPE-containing photoreceptor-less eyecups (Fig. 3d). While 11-*cis*-retin*al* levels are greatly affected by light in the AdipoR1^+/+^ mice, they are not altered in the AdipoR1^−/−^ animals (Fig. 3d). Thus, an impaired visual cycle in AdipoR1^−/−^ mice suggests physiological disturbances that may contribute to photoreceptor degeneration.

### DHA incorporation is reduced in AdipoR1^−/−^ mice

AdipoR1 may promote the key DHA recruitment and retention that, in turn, leads to membrane organization and mediator bioactivity that enable visual function. DHA is transported through the choriocapillaris and is taken up by RPE and photoreceptors, where it is then esterified, elongated and made available on demand to a pool of unesterified (free) DHA that can be converted to bioactive mediators (docosanoids, for example, NPD1).^1^ The liver content of free and total DHA showed no differences between the 12-day-old AdipoR1^+/+^ and ^−/−^ genotypes (Fig. 3e,f). Retinal content of both total and unesterified DHA was examined in mice at 20 days of age. This time point was selected because it represents an age at which there are profound physiological deficits observed in the AdipoR1^−/−^ retina, despite intact anatomy (Figs 1c–g and 2a,b). Remarkably, the content of total DHA was reduced in AdipoR1^−/−^ mice compared with either AdipoR1^+/+^ or AdipoR1^+/−^ mice (Fig. 3g). Unesterified DHA was reduced by 30% and 60% in the AdipoR1^+/−^ and AdipoR1^−/−^ retinas, respectively (Fig. 3h). When the *AdipoR1* gene was absent, there was only minimal unesterified DHA, indicating that AdipoR1 must be present for DHA uptake.

To confirm that AdipoR1 deficiency is associated with compromised DHA uptake and incorporation into photoreceptor OS membranes *in vivo*, labelled DHA (DHA-d5) was delivered systemically to 14-day-old AdipoR1^+/+^, ^+/−^ and ^−/−^ mice, and the retinas were harvested for analysis 6 days later when the mice were 20 days of age. A decrease in total retinal DHA was observed in the AdipoR1^−/−^ mice compared with AdipoR1^+/+^ controls (Fig. 3i). Intermediate levels of DHA-d5 were observed in the AdipoR1^+/−^ mice, indicating a gene dosage effect on retinal DHA uptake and incorporation. These results demonstrate a requirement for AdipoR1 to establish the appropriate concentrations of DHA in photoreceptors.

As mentioned earlier, AdipoR1 is widely expressed in many tissues, such as the liver. Deficiency in RPE/photoreceptor DHA could arise due to compromised packaging and transport of DHA from the diet to the retina. To assess whether the retina is intrinsically defective in DHA uptake and retention, AdipoR1^+/+^, ^+/−^ and ^−/−^ eyecups from 20-day-old mice were incubated with DHA-d5 (4 h, 2 μg). Total and unesterified retinal DHA-d5 displayed ~200 ng mg^−1^ of total protein in AdipoR1^+/+^ eyecups (Fig. 3j). However, total DHA-d5 in AdipoR1^+/−^ and ^−/−^ eyecups was reduced by 50% and 25%, respectively. Moreover, the unesterified DHA pool decreased to minimal levels, indicating incorporation of DHA-d5 (Fig. 3j). Thus, AdipoR1 deficiency hindered the ability of retinal cells to take up and metabolically channel DHA-d5 for esterification.

To determine specificity of the DHA uptake facilitated by AdipoR1, we assessed the endogenous total retinal content of the major member of the other family of essential FAs (omega-6), arachidonic acid, in retinas from 28-day-old mice. We found that total arachidonic acid (free and esterified to phospholipids) remained constant regardless of the genotype (Fig. 3k). These results imply that AdipoR1 sustains DHA content in photoreceptors, and deficiency in AdipoR1 impairs the ability to attain the appropriate levels of this FA in photoreceptor membranes. Moreover, analysis of retinal PC-associated VLC-PUFAs revealed severe reduction in the AdipoR1^−/−^ mice, with almost complete loss of total and unesterified 32:6 and 34:6 (Fig. 3l,m).

### AdipoR1 overexpression or silencing alters DHA in RPE cells

DHA arrives from the blood stream through the choriocapillaris to the RPE cells, which, in turn, transfer the FA to photoreceptor inner segments. Hence, we tested the ability of human spontaneously transformed RPE cells (ARPE-19), a neuroectoderm-derived post-mitotic cell that occurs within the retina, an integral part of the CNS^16^,^17^,^18^, to take up DHA-d5 (100 nM) from the incubation media during a 4-h period. Under resting conditions, these cells display an active ability to incorporate DHA, as evidenced by the reduction in media DHA-d5 to 50% (Fig. 3n). However, unesterified cellular DHA gradually decreased (Fig. 3o), while esterified DHA-d5 greatly increased (Fig. 3p), indicating DHA incorporation into phospholipids. When AdipoR1 was overexpressed in ARPE-19 cells, esterified (incorporated) DHA increased by twofold (Fig. 3q), while esterified DHA-d5 decreased by 20% in silenced ARPE-19 cells (Fig. 3q; the AdipoR1 protein content in silenced and overexpressed ARPE-19 cells is shown in Supplementary Fig. 1). Thus, interconversion of unesterified and esterified DHA occurs within RPE and is enhanced by increased AdipoR1 expression.

### PC-VLC-PUFAs are reduced in AdipoR1^−/−^ retinas

**Figure 4:**
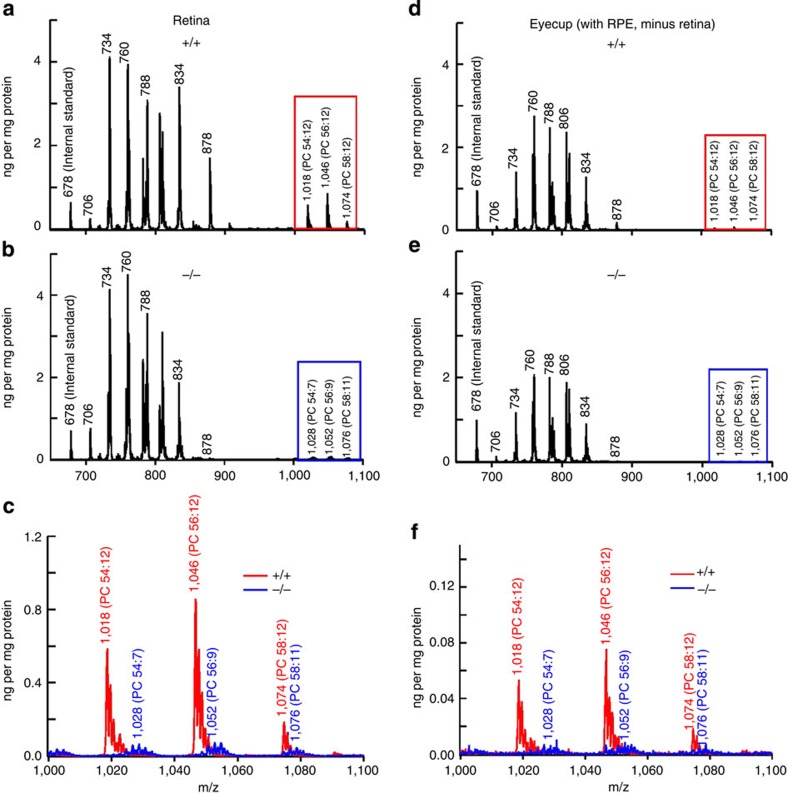
PC molecular species of isolated retinas and RPE cups from 24-day-old ^+/+^ and ^−/−^ mice. Analysis by LC/MS shows similarities among PCs from 700–1,000 *m*/*z*. However, the VLC-PUFAs, ranging from 1,000 to 1,100 *m*/*z* show significant reduction in the AdipoR1^−/−^ retinas, as well as a change in species. Animals of ^+/+^ (**a**,**d**; red boxes) have VLC-PUFA-containing PCs (PC 54:12, PC 56:12 and PC 58:12), whereas ^−/−^ (**b**,**e**; blue boxes) have significantly decreased amounts with different VLC-PUFA-containing PC distributions (PC 54:7, PC 56:9 and PC 58:11). (**c**,**f**) (The red spectra are enlarged from the red boxes above; the blue spectra are from the blue boxes above.) All samples were normalized by the protein and the internal standard PC(28:0), appearing at 678 *m*/*z* within each spectrum.

**Figure 5:**
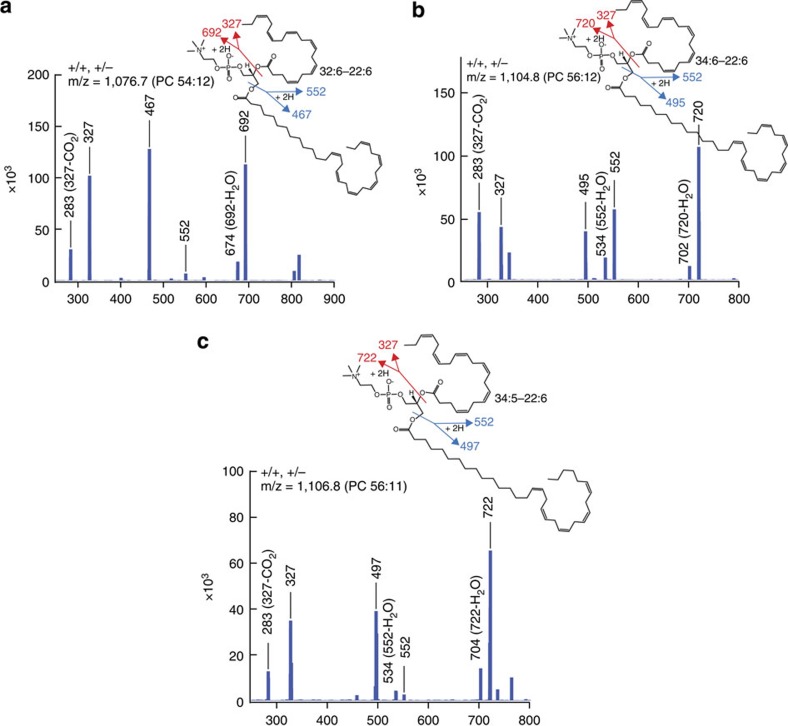
Full fragmentation spectra and structure of AdipoR1^+/+^ and ^+/−^ PC species. The molecular structures of PC54:12, PC56:12 and PC56:11 in 24-day-old mouse retinas were determined from analysis of negative ion mode spectra. (**a**) Spectrum of PC 54:12 (*m*/*z*=1,076.7=M(1,017.7) + Hac (60)—H) shows the fatty acid (FA) composition as FA32:6 and FA22:6. *m*/*z* of 692 is produced from M-(FA22:6-H)+2H, *m*/*z* 552 from M-(FA32:6-H)+2H, *m*/*z* 327 from FA22:6-H and *m*/*z* 467 from FA32:6-H. (**b**) Spectrum of PC 56:12 (*m*/*z*=1,104.8=M(1,045.8) + Hac (60)—H) shows the fatty acid composition as FA34:6 and FA22:6. *m*/*z* of 720 is produced from M-(FA22:6-H)+2H, *m*/*z* 552 from M-(FA34:6-H)+2H, *m*/*z* 327 from FA22:6-H and *m*/*z* 495 from FA34:6-H. (**c**) Spectrum of PC 56:11 (*m*/*z*=1,106.8=M(1,047.8) + Hac (60)—H) shows the FA composition as FA34:5 and FA22:6. *m*/*z* of 722 is produced from M-(FA22:6-H)+2H, *m*/*z* 552 from M-(FA34:5-H)+2H, *m*/*z* 327 from FA22:6-H and *m*/*z* 497 from FA34:5-H. Thus, PC 56:11 is composed of FA22:6 and FA34:5 rather than FA22:5 and FA34:6. (**a**) PC 54:12 is composed of two acyl-fatty acids esterified to sn-1 and sn-2 positions of a glycerol backbone. The spectra in this figure indicate that the composition of the two acyl-fatty acids is FA22:6 (DHA) and FA32:6. The fragmentation places and product *m*/*z* are indicated. (**b**) PC 56:12 is composed of FA22:6 (DHA) and FA34:6. (**c**) PC 56:11 in this sample is composed of FA22:6 (DHA) and FA34:5, rather than from FA22:5 and FA34:6, which would show *m*/*z* of 329, 554, 720 and 499 as product ions, instead. The molecular structures of these three phospholipids accompany each spectrum.

**Figure 6:**
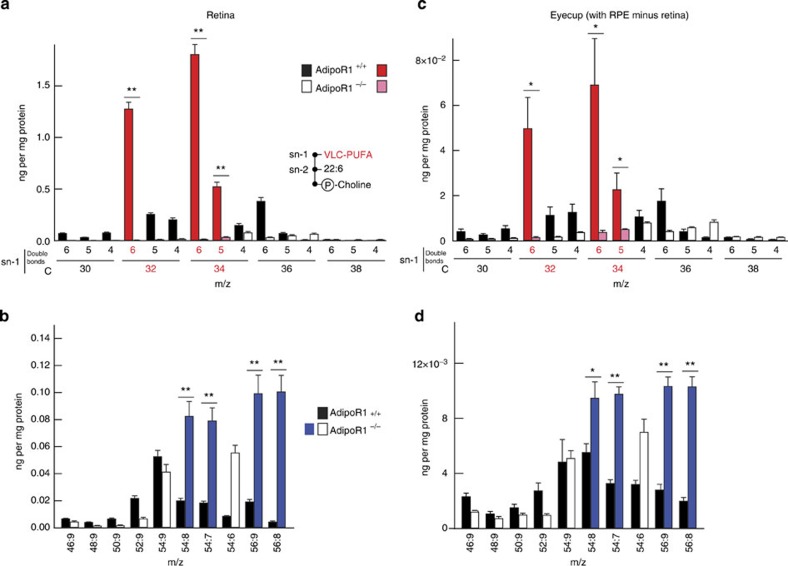
PC species containing VLC-PUFAs are reduced in retinas/RPE cups of AdipoR1^−/−^ mice. Photoreceptor PCs have DHA esterified at the sn-2 position and VLC-PUFAs at sn-1. LC-MS/MS analysis of 24-day-old mouse retina/REP cups shows differences in PC molecular species containing 52–60 carbons. Note that FAs with up to 38 carbons occur at the sn-1 position and 22:6 (22 carbons with six double bonds) is always esterified at the sn-2 position of these retinal PCs. Therefore, only the VLC-PUFAs esterified at sn-1 of these PCs are denoted along the horizontal axis (see inset at lower right in **a** for retinal PC structure). Notice the decline of PCs in the AdipoR1^−/−^ retinas and RPE cups (**a**,**c**). Unusual VLC-PUFA-containing PCs are presented separately. Note that these species increase in the ^−/−^ retinas and RPE cups (**b**,**d**). Some molecules are highlighted in red to correspond to the molecular structures of Fig. 5. The molecules denoted in blue correspond to the ‘odd’ molecular structures of Supplementary Fig. 3. (*n*=5, error bars represent s.e.m., *P<0.05 and **P<0.001, determined by *t*-test.)

PC comprises 43–48% of retinal phospholipids and is richly endowed with DHA^19^. The ELOVL4 is utilized by photoreceptors to specifically elongate DHA to 32–38 carbons (VLC-PUFAs). These VLC-PUFAs, in turn, produce PC molecular species with VLC-PUFAs esterified at sn-1, while DHA (22:6) is esterified at sn-2 (ref. 20). The above results establish that intrinsic AdipoR1 function is required to generate the appropriate levels of DHA in RPE photoreceptors, which is then converted into VLC-PUFAs. These PC-VLC-PUFAs are severely reduced in AdipoR1^−/−^ retinas (Figs 4a–c and 6a), despite the expression of ELOVL4 (Supplementary Fig. 2). Full fragmentation spectra of PC(54:12), PC(56:12) and PC(56:11) from AdipoR1^+/+^ and ^+/−^ retinas revealed their molecular structure (Fig. 5). The FA compositions of PC(54:12), PC(56:12) and PC(56:11) are FA32:6 and FA22:6, FA34:6 and FA22:6, and FA34:5 and FA22:6, respectively, that is, a VLC-PUFA and DHA(22:6). The ‘odd’ PC species present in the AdipoR1^−/−^ retinas were very low in abundance. However, the full MS fragmentation spectra and molecular structures of PC(54:8), PC(54:7), PC(56:9) and PC(56:8), some of the more abundant species, do not contain 22:6 and are mixtures of FAs (Fig. 6b and Supplementary Fig. 3), indicated by the presence of additional mass numbers not part of the illustrated molecule (for example, *m*/*z* 496 and 704 in PC(54:8)). Only the more prevalent molecular species are shown.

**Figure 7:**
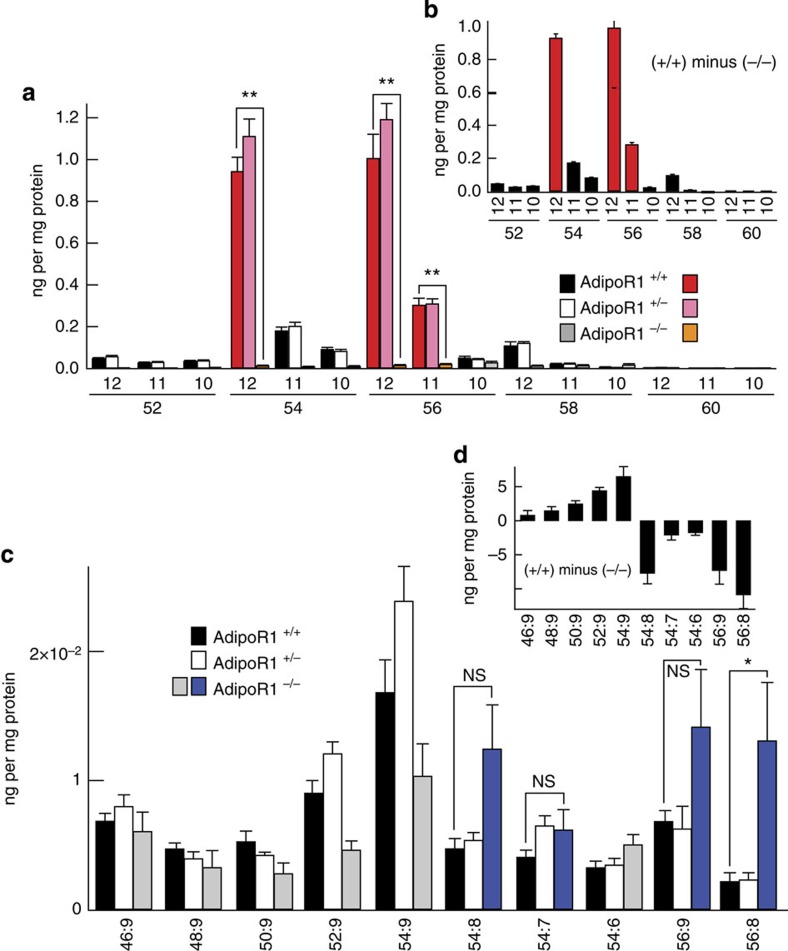
PC VLC-PUFA species of isolated retinas from 15-month-old ^+/+^, ^+/−^ and ^−/−^ mice. (**a**) The PC-containing VLC-PUFA profile (22:6 is esterified at the sn-2 position) of old AdipoR1^+/+^ mice is similar to that of young animals, but is greatly diminished in the old AdipoR1^−/−^ retinas. (**b**) The difference profile (the ^+/+^ minus the ^−/−^ profile) represents photoreceptor-specific VLC-PUFAs. (**c**) Unusual VLC-PUFA-containing PCs increase in the ^−/−^ retinas. (**d**) The difference profile (the ^+/+^ minus the ^−/−^ profile) of the unusual VLC-PUFAs reveals photoreceptor-specific (positive-going) and retina-specific (negative-going) PC species. Red bars indicate molecules that are diagrammed in Fig. 5, Blue bars indicate molecules that are diagrammed in Supplementary Fig. 3. In AdipoR1^+/+^, *n*=7; AdipoR1^+/−^, *n*=6; AdipoR1^−/−^, *n*=6. Error bars represent s.e.m., *P<0.05, and **P<0.001, determined by *t*-test. NS=nonsignificant *P* value.

**Figure 8:**
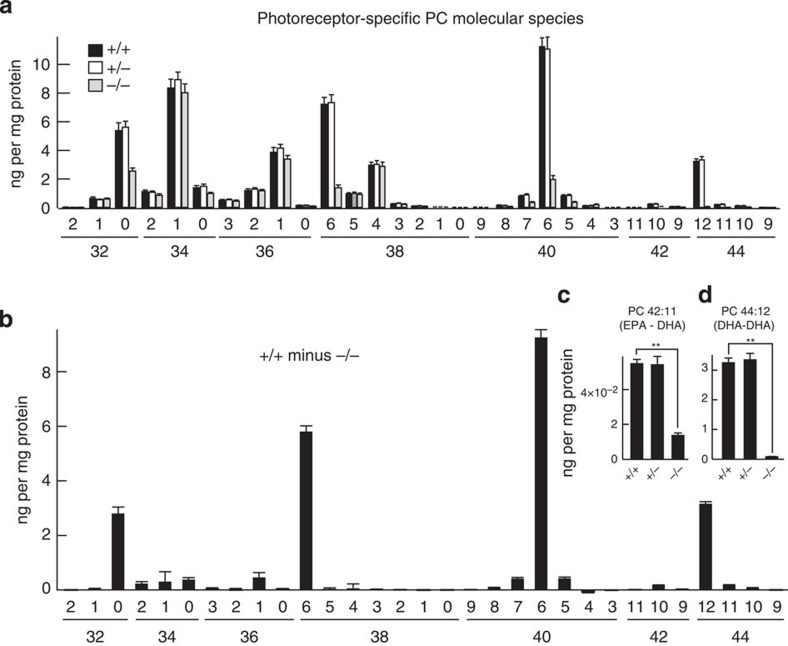
Photoreceptor-specific PC species of retinas of 15-month-old ^+/+^, ^+/−^ and ^−/−^ mice. (**a**) Profile of the short-chain PCs shows similar amounts in the ^+/+^ and ^+/−^ retinas, but diminished values for several PC species in the ^−/−^ animals. (**b**) The difference profile from the ^+/+^ and ^−/−^ retinas reveals four highly enriched photoreceptor PC species. Each of these, except 32:0, also contains 22:6. (**c**) PC42:11 (eicosapentaenoic acid, EPA-DHA) and (**d**) PC42:12 (DHA–DHA) from 24-day-old mice revealed similar quantification between AdipoR1^+/+^ and ^+/−^ retinas, but greatly reduced amounts within the AdipoR1^−/−^ genotype. (In **a**,**b**, AdipoR1^+/+^, *n*=7; AdipoR1^+/−^, *n*=6; AdipoR1^−/−^, *n*=6. In **c**,**d**, AdipoR1^+/+^, *n*=17; AdipoR1^+/−^, *n*=17; AdipoR1^−/−^, *n*=11. Error bars represent s.e.m., and **P<0.001, determined by *t*-test.)

**Figure 9:**
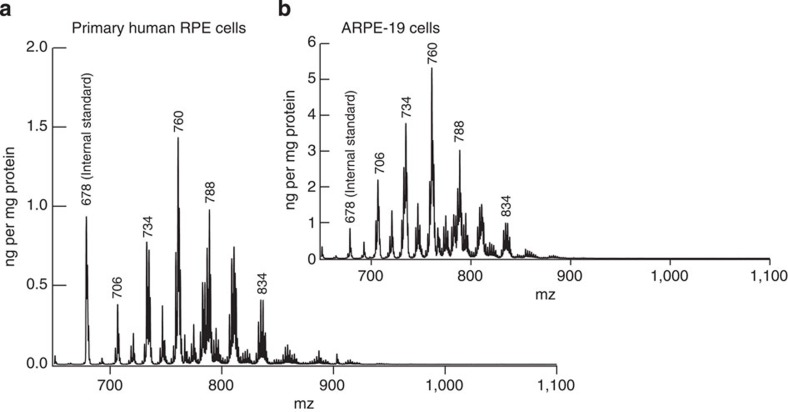
Phosphatidylcholine molecular species of ARPE-19 and primary human RPE cells. ARPE-19 and the primary human RPE cells also exhibit a PC profile from 700 to 900 *m*/*z* that is similar to retinal and RPE profiles, but completely lack PCs within the 1,000–1,100 *m*/*z* VLC-PUFA range, indicating that RPE does not synthesize VLC-PUFAs.

RPE cups (eyecups with RPE minus retina) from AdipoR1^+/+^, ^+/−^ and ^−/−^ mice, as well as the dissected retinas from the respective RPE cups, were used to isolate, characterize and quantify VLC-PUFAs by LC-MS/MS tandem mass spectrometry. Both retina (with photoreceptors) and RPE cups (containing only the RPE cells) demonstrated VLC-PUFAs of up to 38 carbons. Comparisons between the ^+/+^ and ^−/−^ genotypes for 24-day-old mice revealed similar PC molecular species profiles from the ^+/+^ and ^−/−^ mice from 700 to 1,000 *m*/*z*, but all VLC-PUFA PC molecules (1,000–1,100 *m*/*z*) were greatly reduced in the retinas of the ^−/−^ mice and were of different species (Figs 4a–c and 6a,b). Importantly, these unusual PC molecular species that were very rare in wild-type animals were produced in the ^−/−^ retinas (Figs 4c and 6b). Similar profiles were found in retinas of 15-month-old mice (Fig. 7a,c). This suggests that retinas lacking AdipoR1 do not have the ability to produce the VLC-PUFAs, regardless of age. PC profiles from the 24-day-old RPE cups were similar to those of their corresponding retinas (Figs 4d–f and 6c,d). Phospholipid profiles of the short-chain FA-containing PCs were also obtained from the 15-month-old mouse retinas, demonstrating abundance of 32:0, 34:1, 36:1, 38:6, 38:4, 40:6 and 44:12 (Fig. 8a). AdipoR1^+/+^ and ^+/−^ retinas were remarkably similar, but the ^−/−^ retinas showed extensive reductions in 32:0, 38:6, 40:6 and 44:12. Because the photoreceptors had degenerated in these old ^−/−^ mice, we generated a difference profile by subtraction from the photoreceptor-rich ^+/+^ mouse profile to generate a photoreceptor-specific PC profile (Fig. 8b). The ^+/+^ mouse photoreceptors are highly enriched in 32:0, 38:6, 40:6 and 44:12. When a difference profile was generated for photoreceptors from the ^+/+^ and ^−/−^ retinas for the 22:6-containing VLC-PUFA PCs, in addition to the presence of 22:6 at the sn-2 position, 32:6 and 34:6 were very abundant and 32:5, 32:4, 34:5 and 36:6 were also prevalent (Fig. 7b). Interestingly, the difference profile between the ^+/+^ and ^−/−^ retinas of the unusual VLC-PUFAs (Fig. 7c,d) revealed the prevalence of 46:9, 48:9, 50:9, 52:9 and 54:9 photoreceptor-specific PCs (positive-going bars) and 54:8, 54:7, 54:6, 56:9 and 56:8 remaining retina-specific PCs (negative-going bars). Since the ^−/−^ retinas had lost most photoreceptors, the appearance of these remaining PCs originated from the inner retina, neuronal or Müller cell remodelling, or the influx of monocytes/macrophages and the possible expression of microglial protective/damaging phenotypes. However, a single row of highly ordered nuclei remained within the ONL at 1 year of age in AdipoR1^−/−^ (Fig. 1f,g), suggesting the survival of cone photoreceptors. Thus, these retina-specific PCs (negative-going bars) may represent a cone profile. Lipidomic analysis has tentatively identified the fatty acyl chains of 54:8 (Fig. 7d) as FA20:4 (arachidonic acid, omega-6) and FA34:4 (Supplementary Fig. 3a). With the absence of AdipoR1, and, consequently, greatly reduced 22:6, compensatory mechanisms may take over. A shift from an omega-3-driven synthesis of VLC-PUFAs, in conjunction with 22:6, to an omega-6-based synthesis that substitutes 20:4 for 22:6 and produces omega-6 VLC-PUFAs, may result. Further analysis of PC42:11 (EPA, 20:5n3-DHA) and PC44:12 (DHA-DHA) revealed no differences between the AdipoR1^+/+^ and ^+/−^ retinas, but showed extensive loss in the AdipoR1^−/−^ retinas (Fig. 8c,d). There was a hundredfold decrease in DHA–DHA-containing species, but only a 10% loss in EPA–DHA-containing species. In addition, the ratio of free EPA to EPA-d5 was 0.27 in AdipoR1^+/+^ and 0.24 in AdipoR1^−/−^ retinas, indicating no significant change in free EPA. This demonstrates no change in retinal EPA. Thus, since EPA is a precursor for the VLC-PUFAs, but is unchanged, DHA may also be a substrate for ELOVL4 directly or by conversion into EPA. This step is known to require peroxisomes, and their possible presence in photoreceptors should be ascertained. Sphingomyelins also contain VLC-PUFAs, and, thus, might be affected by the lack of AdipoR1. However, when retinal sphingomyelin from the ^+/+^ and ^−/−^ genotypes was compared, there was no difference (Supplementary Fig. 4). When analyses of ARPE-19 and human primary RPE cells were performed, no VLC-PUFAs were detected (Fig. 9). This suggests that VLC-PUFAs within the RPE cups (Figs 4d–f and 6c,d) occurred as a result of shedding and phagocytosis of photoreceptor apical disk membranes, which are richly endowed with VLC-PUFAs. The ARPE-19 cells and the tested human primary RPE cells had not undergone phagocytosis; therefore, they were devoid of these FAs. Thus, RPE cells do not synthesize VLC-PUFAs, but likely contribute to their retention in PRCs by sending them back to the inner segments as they do with DHA.

## Discussion

**Figure 10:**
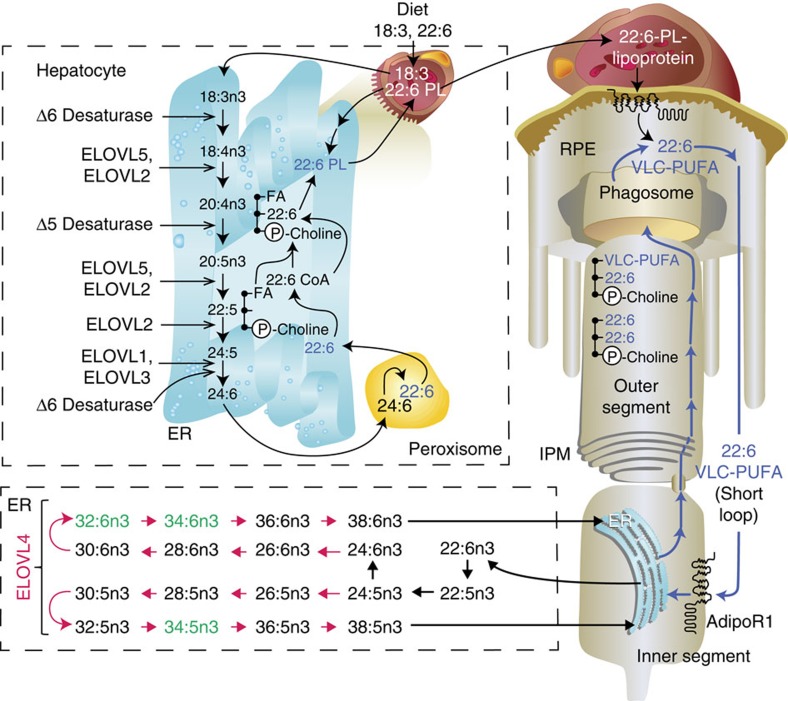
Biosynthesis pathway for the omega-3 fatty acids. Diagram depicting the desaturation and elongation steps in the generation of VLC-PUFAs as these molecules traffic through the endoplasmic reticulum and the peroxisome of the hepatocyte, the endoplasmic reticulum of the photoreceptor inner segment and into the photoreceptor outer segment. The elongation steps catalysed by ELOVL4 are highlighted in red. RPE retrieval of DHA (22:6) and of the VLC-PUFAs from shed photoreceptor apical disk membranes is followed by recycling of DHA and of the VLC-PUFAs back to the photoreceptor inner segment. C, carbons; ELOVL, elongase of the very long-chain fatty acids; ER, endoplasmic reticulum; IPM, interphotoreceptor matrix; PL, phospholipid; RPE, retinal pigment epithelium.

The essential omega-3 FA, DHA, is transported from the liver to the RPE cells, from which it is taken up by the inner segments of PRCs, esterified into phospholipids and used for the biogenesis of PRC membranes as a major acyl chain of OS disk membranes (Fig. 10)^1^. PC in PRCs comprises half of the retinal phospholipids and is richly endowed with DHA that includes supraenoic molecular species containing two docosahexaenoyl chains per molecule^21^ as well as with VLC-PUFAs^22^. ELOVL4 elongates DHA to 32–38 carbons (VLC-PUFAs) in PRCs. After activation to acyl-Co A, these FAs are selectively acylated at sn-1 of PC molecular species, whereas DHA is esterified at sn-2 (ref. 20).

Mutations in ELOVL4 are causative of autosomal dominant Stargardt-like macular dystrophy 3 (STGD3; OMIM no. 600110). Mutated ELOVL4 is associated with severe reduction in the levels of VLC-PUFAs^23^,^24^, homo- and/or hetero-oligomerization of a truncated ELOVL4ΔC with other elongases^25^, and intracellular accumulation of the mutant ELOVL4 (ref. 26). Inappropriate localization of mutant ELOVL4 to rod OSs was recently demonstrated in the *Xenopus* retina^27^. Deficiency of VLC-PUFAs alone may not be the key driver of photoreceptor loss in the AdipoR1 KO because photoreceptor structure and function is maintained in rod or cone conditional KOs of ELOVL4 (ref. 28). Moreover, ELOVL4 is expressed in the AdipoR1 KO retina and we discovered rare lipid species, suggesting the photoreceptors/retina attempt to compensate for deficiencies in DHA and VLC-PUFA. It is of interest that a shortage in VLC-PUFAs has also been reported in AMD^29^. Further studies of VLC-PUFAs in macular dystrophies and degeneration may reveal new insights into the cause of photoreceptor and retinal pigment epithelial cell death.

The precise mechanisms that underlie the acquisition and retention of DHA by the CNS are unclear. Recently, an essential role for Mfsd2a, a member of the major facilitator superfamily, was shown to mediate DHA transport in the form of a lysophospholipid across the blood brain barrier^30^. However, KO of Mfsd2a does not lead to retinal degeneration^30^. The ablation of AdipoR1 has allowed us to discover that this receptor regulates DHA levels in the retina. Moreover, the DHA changes are selective for the essential omega-3 FA family, since arachidonic acid content (from the essential omega-6 FA family) remained unchanged in AdipoR1^−/−^ retinas. AdipoR1 promotes DHA uptake that enables its conversion to VLC-PUFA. Photoreceptor cell-specific PC molecular species containing VLC-PUFAs are greatly reduced in AdipoR1^−/−^. VLC-PUFAs, after shedding and phagocytosis of photoreceptor apical disk membranes, appear transiently in RPE cells, suggesting that RPE cells do not have the intrinsic ability to biosynthesize VLC-PUFAs. As RPE retrieves DHA through the interphotoreceptor matrix short loop to the PRC inner segment^31^,^32^,^33^, the VLC-PUFAs may also follow a similar route from the shed photoreceptor apical disk membranes, which permits recycling of this essential omega-3 FA and the VLC-PUFAs back to the PRC inner segment (Fig. 10). Under conditions of uncompensated oxidative stress, or other homeostatic disruptions, DHA is released from membrane phospholipids and converted to NPD1, a docosanoid mediator that promotes photoreceptor^34^ and RPE^35^ cell survival^1^. The role of VLC-PUFAs as mediators of cell signalling and/or survival remains to be tested. Our work identifies AdipoR1 as a key regulator of PRC survival through DHA uptake, retention, conservation and elongation to VLC-PUFAs. Thus, the photoreceptor-RPE/DHA lipidome endogenous cell survival responses offer mechanisms that could be mimicked to therapeutically counteract early stages of retinal-degenerative diseases.

## Methods

All procedures have been carried out according to the Association for Research in Vision and Ophthalmology statement for the Use of Animals in Ophthalmic and Vision Research and the Louisiana State University Health Sciences Center, New Orleans, Institutional Animal Care and Use Committee. Throughout this study, male mice were used exclusively with the exception of the experiments in Fig. 3k–m. Here the gender distribution was: AdipoR1^+/+^, males=11, females=6; AdipoR1^+/−^, males=8, females=9; AdipoR1^−/−^, males=5, females=6.

### Generation of AdipoR1^gt^ mice

The generation of the OmniBank gene trap library has been described^36^,^37^. The AdipoR1 mutant mice were generated by microinjection of embryonic stem (ES) cells cloned into host blastocysts using standard described methods^38^,^39^. Mouse ES cells carrying a mutation in the *AdipoR1* gene (accession number NM_028320) were obtained from OmniBank, a sequence-tagged library of gene-trapped ES cell clones. An ES cell clone with an insertion in intron 5 of mouse *AdipoR1*, downstream of the initiation codon in exon 2, was selected to generate mice heterozygous for a potential null allele of the gene (Supplementary Fig. 5a). Interbreeding of AdipoR1^+/−^ mice gave rise to the expected Mendelian ratios of wild-type, heterozygous and homozygous animals. To confirm the disruption of *AdipoR1* transcription by the gene-trapping vector, we performed reverse transcription–PCR (RT–PCR) using primers complementary to *AdipoR1* exons 5 and 6, flanking the site of integration of the vector (Supplementary Fig. 5b). Wild-type *AdipoR1* transcript was not detected in tissues of *AdipoR1*^*−/−*^ mice (Supplementary Fig. 5c). The precise genomic insertion site of the retroviral gene-trapping vector in the *AdipoR1* gene was determined by inverse genomic PCR as previously described^40^.

### Generation of mutant AdipoR1^hr^ mice

The conditional AdipoR1-targeting vector was derived using the Lambda KOS system^41^. The Lambda KOS phage library, arrayed into 96 superpools, was screened by PCR using exon 3-specific primers AdipoR1–6 (5′- GTCTAGGCTTGGTGCACTAAG -3′) and AdipoR1–5 (5′- GCAAGTGCTCTTCAACTCCAG -3′). The PCR-positive phage superpools were plated and screened by filter hybridization using the 491-bp amplicon derived from primers AdipoR1–6 and AdipoR1–5 as a probe. Two pKOS genomic clones, pKOS-61 and pKOS-54, were isolated from the library screen and were confirmed by sequence and restriction analysis. Gene-specific arms (5′- ATTTAAACAATGAGCTAGCCTTTACAGTTAGA -3′) and (5′- AGTGCCGGAACTAAAGGAGTGCATCGCCACCA -3′) were appended by PCR to a yeast selection cassette containing the URA3 marker. The yeast selection cassette and pKOS-54 were co-transformed into yeast, and clones that had undergone homologous recombination to replace a 2,166-bp region containing exons 2–4 with the yeast selection cassette were isolated. This 2,166-bp fragment was independently amplified by PCR and cloned into the intermediate vector pLFNeo introducing flanking LoxP sites and a Neo selection cassette (AdipoR1-pLFNeo). The yeast cassette was subsequently replaced with AdipoR1-pLFNeo selection cassette to complete the conditional AdipoR1-targeting vector that has exons 2–4 flanked by LoxP sites. The NotI linearized targeting vector was electroporated into 129/SvEv^Brd^ (Lex-1) ES cells. G418/FIAU-resistant ES cell clones were isolated, and correctly targeted clones were identified and confirmed by Southern blot analysis using a 538-bp 5′ external probe (20/21), generated by PCR using primers AdipoR1–20 (5′- GTACTCAATAGATGCTCTCAG -3′) and AdipoR1–21 (5′- AGTTTGTACAGCATAGCACTC -3′), and a 354-bp 3′ internal probe (23/22), amplified by PCR using primers AdipoR1–23 (5′- GCAAAATGTTCTCAACTTTGAGG -3′) and AdipoR1–22 (5′- CCCACTGTGCCACAATGATG -3′). Southern blot analysis using probe 20/21 detected an 8.5-kb wild-type band and a 10.4-kb mutant band in BamHI-digested genomic DNA, while probe 23/22 detected a 7.9-kb wild-type band and a 5.9-kb mutant band in KpnI-digested genomic DNA. Two targeted ES cell clones were identified and microinjected into C57BL/6J (albino) blastocysts to generate chimeric animals that were bred to C57BL/6J (albino) females, and the resulting heterozygous offsprings were interbred to produce homozygous conditional AdipoR1-deficient mice. Determination of the genotype of mice at the AdipoR1 locus was performed by screening DNA from tail biopsy samples using quantitative PCR for the *Neo* cassette. The Neo primers used to genotype the mice were: 5′- CTCCTGCCGAGAAAGTATCCA -3′, 5′-GGTCGAATGGGCAGGTAG-3′ and 5′-6FAM- ATGGCTGATGCAATGCGGCG -TAMRA-3′. This strategy enabled the discrimination of zero, one or two conditional gene disruptions representing AdipoR1^+/+^, AdipoR1^+/−^ and AdipoR1^−/−^ mice, respectively. Deletion of the floxed region spanning exons 2–4 was accomplished by crossing this line to a Protamine-Cre recombinase-expressing line^42^. Retinal phenotypes were indistinguishable between the AdipoR1^gt^ and ^hr^ alleles; therefore, all data in this manuscript are from the AdipoR1^gt^ allele.

### AdipoR1 RT–PCR

RNA was extracted from the kidney and spleen using a bead homogenizer and RNAzol (Ambion) according to the manufacturer’s instructions. Reverse transcription was performed with SuperScript II (Invitrogen) and random hexamer primers, according to the manufacturer’s instructions. PCR amplification (95 °C, 30 s, 59 °C, 45 s, 70 °C, 60 s) was performed for 35 cycles using primers complementary to exons 5 and 6 of the *AdipoR1* gene, flanking the insertion site of the vector (Primer C: 5′- GAGAAGGTGGTCTTCGGGATGTTCTT -3′ and 5′- GGCTGTGGGGAGCAGTAGAAGGAGTAATAG -3′). Control primers to the mouse β*-Actin* gene (accession number M12481) were: 5′- GGCTGGCCGGGACCTGACGGACTACCTCAT -3′ and 5′- GCCTAGAAGCACTTGCGGTGCACGATGGAG -3′. *AdipoR1* RT–PCR products were verified by sequencing.

### *In situ* hybridization

*In situ* hybridization analysis was performed on 16-μm cryosections of eyes obtained from 2-month-old mice, as described elsewhere^43^. An *Adipor1*-specific cDNA (base pairs 718–914 of NM_028320) was generated by PCR with primers that incorporate the T7 RNA polymerase promoter sequence into the PCR amplicon. This DNA template was used for *in vitro* transcription reaction with 80 μCi of αP33-UTP (NEN Life Science Products, Boston, MA). After hybridization at 60 °C for 16 h, sections were treated with RNase and washed in SSC buffer. Slides were dehydrated in a graded ethanol series and exposed to a 50% solution of autoradiographic emulsion type NTB2 (Eastman Kodak Company, Rochester, NY) for 3–6 days. Slides were developed using standard protocols^44^, dehydrated and coverslips were applied (Permount; Fisher Scientific, Pittsburgh, PA). Digital images were acquired (ORCA II; Hamamatsu) using a cooled CCD camera mounted on a BX60 (Olympus) microscope equipped with dark-field optics.

### Characterization of AdipoR1^−/−^ mice

Histopathology was conducted on mice at various ages from postnatal day 14 to 41 weeks of age. Mice were killed by CO_2_ asphyxiation and their eyes were removed and placed in Davidson’s fixative (Poly Scientific; Bay Shore, NY) overnight at room temperature. For TUNEL staining (DeadEnd Fluorometric TUNEL System; Promega, Madison, WI), eyes were processed into paraffin, sectioned at a thickness of 6 μm and the TUNEL reaction performed according to the manufacturer’s instructions. For histopathology analysis, tissues were collected from KO mice and age-matched wild-type mice, fixed in either modified Davidson’s fixative (Polysciences Inc) or 10% neutral buffered formalin, and sections similarly prepared, followed by haematoxylin and eosin staining for light microscopy and imaging.

### OCT

AdipoR1 and Adiponectin KOs were anaesthetized with a mixture of ketamine/xylazine (200 and 10 mg kg^−1^, i.p.), placed in a conical tube with the tip removed to expose the nose and eyes, and wrapped in a warming blanket. Then, the eyes were dilated with tropicamide, followed by placement of noncorrecting contact lenses to prevent corneal desiccation. Retinas were imaged with SD-OCT along the vertical meridian through the optic disc (superior retina and inferior retina) using a Heidelberg Spectralis HRA+OCT system (Heidelberg Engineering, Heidelberg, Germany). Axial resolution is 7 mm optical and 3.5 mm digital. The SD-OCT imaging was conducted at five time points (3, 5, 9, 16 and 33 weeks of age). The methodology provided here has also been described in detail previously^45^,^46^.

### ERGs

ERGs were recorded from wild-type (*n*=4) and homozygous KO (*n*=6) mice at 3–4 weeks of age under dim red light (>650 nm) from both eyes simultaneously by an electrodiagnostic system (Espion E^2^, Diagnosys LLC, Lowell, MA). After overnight dark adaptation, mice were anaesthetized with an ip injection cocktail (10 ml kg^−1^ body weight) containing (per ml) ketamine (7.5 mg), xylazine (0.38 mg) and acepromazine (0.074 mg). Pupils were dilated with 0.1% atropine (Alcon Laboratories Inc., Fort Worth, TX), and 0.5% proparacaine (Alcon) was applied for topical anaesthesia. Gold electrodes were placed on the corneal surface with a drop of methylcellulose, a reference electrode was placed subcutaneously on the head and a ground electrode was placed in the right hind leg. The animals were positioned in a Ganzfeld illumination dome (ColorDome, Diagnosys LLC). Full-field scotopic ERGs of both eyes were elicited simultaneously with 5-ms light flashes. Routinely, five recordings per flash intensity were averaged across both eyes. Mixed rod–cone (scotopic)-driven responses to light flashes were recorded, with the intervals between flashes increasing from 5 to 250 s, with increasing flash intensity in the range 0.001–2,000 cd s m^−2^.

For analysis of a-wave recovery from bleaching light, 4- to 6-week-old AdipoR1^+/+^ (*n*=5) and AdipoR1^−/−^ (*n*=6) mice were dark-adapted overnight. The following morning, anaesthetized mice were placed on a heating pad at 37 °C for the duration of the ERG recordings. Baseline a-wave responses were recorded following a flash of 0.08 cd s m^−2^ to establish a pre-bleach reference. Mice were then exposed to white light at 400 lux for 5 min and returned to darkness. a-wave amplitudes were then measured every 10 min following a stimulus flash of 0.08 cd s m^−2^. The recording stopped after 60 min of measurement. Data are represented as percent a-wave recovery at each 10-min interval.

### Lipid extraction and LC-MS/MS-based lipidomic analysis

Samples were homogenized in MeOH (3 ml) followed by addition of CHCl_3_ (6 ml) containing an internal standard mixture of AA-d8, PGD2-d4, EPA-d5, 15HETE-d8, LTB4-d4 and PC(28:0) sonicated in an ice water bath and then stored at −80 °C overnight. After vortexing and centrifugation, the supernatant was removed and the pellet washed with CHCl_3_/MeOH (1 ml, 2:1), centrifuged and the supernatants combined. Distilled water (2 ml, pH 3.5) was then added to the supernatant, centrifuged and the pH of the upper phase adjusted to 3.5–4.0 with HCl. The lower phase was then dried down under N_2_, resuspended in methylformate (200 μl), transferred to a vial, dried under N_2_, resuspended in MeOH (10 μl) and water (5 μl) added. To determine total DHA, AA and their derivatives, samples were hydrolysed by incubating in a water bath with NaOH at 42 °C for 3 h. Ethylacetate (2 ml) was then added and samples centrifuged, the upper phase isolated and dried under N_2_ and resuspended in MeOH/H_2_O (50 μl, 2:1).

Xevo TQ-S equipped with Acquity I Class UPLC (Waters) was used for liquid chromatography–mass spectrometry (LC-MS/MS) analysis. An Acquity UPLC HSS T3 1.8-μm 2.1 × 50 mm column was used for FAs and their derivatives. Solvent A (75%; H_2_O+0.1% acetic acid) and 25% of solvent B (90% acetonitrile, 10% isopropanol) with a 0.6-ml min^−1^ flow rate was used for the first minute, then graduated to 100% of solvent B for 7.5 min, followed by 100% solvent B for 2.5 min. The column was then re-equilibrated to 75% A and 25% B for 2 min. The capillary voltage was −2.5 kV, the desolvation temperature was set at 600 °C, the desolvation gas flow set to 1,100 l h^−1^, the cone gas at 150 l h^−1^ and nebulizer pressure set at 7.0 Bars, with the source temperature at 150 °C.

For phospholipid molecular species analysis, an Acquity UPLC BEH Amide 1.7-μm 2.1 × 100 mm column was used with solvent A (acetonitrile:water, 1:1; 10 mM ammonium acetate pH 9.1) and solvent B (acetonitrile:water, 95:5; 10 mM ammonium acetate pH 9.1) as the mobile phase. Solvent B (100%) ran for the first 5 min isocratically, was graduated to 20% solvent A for 8 min and then run at 65% of A for 0.5 min. It ran isocratically at 65% of A for 3 min, and then returned to 100% of B for 3.5 min for equilibration. The capillary voltage was 2.5 kV, desolvation temperature was set at 550 °C, the desolvation gas flow rate was 800 l h^−1^, cone gas at 150 l h^−1^ and nebulizer pressure was 7.0 Bars, with the source temperature at 120 °C. For analysis of the phosphatidyicholine molecular species, we used PC(28:0) as our internal standard for normalization.

Lipid standards (Cayman Chemical Company, Ann Arbor, MI) were used for tuning and optimization, and to create calibration curves. Samples were run at least four times, and the concentration curves and one-point comparison method were applied. The usual ratio between unesterified and total endogenous DHA normally is >10,000. Therefore, we used this criterion to ensure that the tissue was properly manipulated and that the extraction and measurements were correct.

### Assessment of retinoid content and visual cycle function

Overnight dark-adapted wild-type (*n*=4) and AdipoR1^−/−^ (*n*=4) mice (3–4 months of age) were anaesthetized with an intraperitoneal injection of ketamine and xylazine. Pupils were dilated with 1% (w/v) atropine sulfate in saline solution. Two mice of each genotype were exposed to light at 1,000 lux for 10 min; the remaining mice were maintained in darkness. Mice were killed by cervical dislocation under anaesthesia and eyes enucleated. After removing the anterior segment, eyecups were homogenized in 20 mM HEPES buffer containing 0.1% SDS and hydroxylamine, and retinoids extracted with hexane under dim red light (Kodak Wratten 1A). Total retinoids, all-*trans*-retinyl esters and 11-*cis* retinaldehyde from the dark-adapted and light-exposed mice were measured by normal-phase HPLC (Agilent 1100 liquid chromatograph) equipped with a ultraviolet photodiode-array detector. Retinoids in the samples were separated by gradient elution of the mobile phase (0.2–10% dioxane in hexane, 2 ml min^−1^ flow rate) on a silica column (Zorbax-Sil 5 μm, 250 × 4.6 mm, Agilent Technologies). Identified peaks were confirmed by spectral analysis and co-elution with authentic retinoid standards.

### Eyecup organotypic cultures

Eyes of AdipoR1^+/+^, AdipoR1^+/−^ and AdipoR1^−/−^ mice were collected and the anterior section removed. The resulting eyecups were incubated for 2 h in 500 μl of DMEM/F12 (1:1) media (Gibco, Grand Island, NY) and 10% FBS containing 20 ng of DHA-d5, and then carefully washed twice with ice-cold PBS (pH 7.4) and homogenized in 1 ml of methanol/water (2:1) and lipid extracted, followed by hydrolysis using methanol and sodium hydroxide at 42 °C for 3 h. Total protein was precipitated and measured using the Lowry method.

### Cell culture and transient transfectants

ARPE-19 cells were maintained in DMEM/F12 (Thermo Scientific, Kalamazoo, MI) containing 10% FBS (Tissue Culture Biologicals, Tulare, CA) and 100 U ml^−1^ of penicillin/streptomycin (Thermo Scientific), as previously described^47^. Cells were incubated at 37 °C with 5% CO_2_ and 99% humidity. Then, cells were transfected with Lipofectamine2000 following the manufacturer’s directions with only slight differences^48^. Cells were plated and incubated for 48–72 h to reach confluency. DHA-d5 (100 nM) was added to the media and the cells harvested at 4 h. Lipids were extracted as described above. Cells were silenced using commercially obtained shRNA-containing plasmids (SABioscience, Valencia, CA). The overexpression was performed using *Mus musculus* AdipoR1.The percentages of silencing and overexpression obtained were 38 and 81% relative to control (Supplementary Fig. 1a,b).

### Immunohistochemistry

Immunolocalization was performed on 20-μm-thick cryosections of 23-day-old mouse eyes. Eyes were fixed in 4% fresh paraformaldehyde in PBS, followed by cryoprotection in 10, 20 and then 30% sucrose. Eyes were oriented and embedded in OCT Compound (Tissue-Tek, Sakura Finetek; Torrance, CA) just before sectioning. Sections were placed on glass microscope slides on a 32 °C slide warmer for 30 min, then rinsed, post-fixed with methanol/acetone (1:1) and permeabilized with 1% triton X-100. After blocking for 1 h in 2% donkey serum, primary antibody was added for 24 h at 4 °C. This was followed by incubation with the secondary antibody and nuclear staining for 1 h at room temperature. Sections were then washed, coverslipped and imaged. The primary antibody was a rabbit polyclonal anti-ELOVL4 at 1:200 dilution. This antibody was a gift from Dr R.E. Anderson (University of Oklahoma Health Sciences Center, Departments of Ophthalmology and Cell Biology, Oklahoma City, OK). The secondary antibody was AlexaFluor 488 goat anti-rabbit IgG (Invitrogen, Carlsbad, CA), applied at 1:200 for 1 h. Nuclei were stained with 10 μg ml^−1^ DAPI. Imaging was performed on a Zeiss LSM-710 Meta laser confocal microscope with a × 20 objective.

### Western blot

Briefly, cells were lysed with RIPA buffer, 30 μg of total protein were placed in Laemmli loading buffer for 5 min at 95 °C and loaded on a NuPAGE precast gels. Gels were run in an X-Cell running system (Invitrogen) with MOPS buffer. Transference took place in a Turbo Transfer system (Bio-Rad, Hercules, CA) following the manufacturer’s instructions. Anti-ELOVL4 (1 μg ml^−1^), 1 μg ml^−1^ anti-Adiponectin Receptor 1 (Abcam, Cambridge, MA) and 200 ng ml^−1^ anti-GAPDH (Millipore, Billerica, MA) primary antibodies were hybridized for 16–48 h at 4 °C, washed and then incubated for 1 h with the Cy3 and Cy5 (Abcam; 400 ng ml^−1^)-coupled secondary antibody at room temperature. Membranes were developed using GE gel documenter (GE Healthcare) following the manufacturer’s instructions.

Retinas from 23-day-old mice were homogenized at 4 °C using a custom buffer consisting of 20 mM Tris-HCl (pH 7.5), 150 mM NaCl, 1 mM EDTA, 1 mM EGTA and 1% Triton and Complete inhibitor (Roche Diagnostics, Indianapolis, IN). Samples were centrifuged 10 min at 14,000 *g*. Protein concentration was determined with a Bradford assay (Bio-Rad). The supernatant was used for sodium dodecylsulfate (SDS)–PAGE and the pellet stored at −20 °C. Equal amounts of nonboiled retinal protein (30 μg per lane) were diluted with SDS sample buffer, loaded on gels for SDS–PAGE (4–12% gradient; Invitrogen, San Diego, CA), and electrophoresed at 125 V for 1.5 h on ice. Proteins were electroblotted to polyvinylidene difluoride membranes using Iblot (Invitrogen). Gel retention was assessed by staining with Coomassie blue (Pierce, Rockford, IL). Nonspecific binding was blocked for 1 h at room temperature with 5% BSA in Tween-Tris-buffered saline (TTBS). The membrane was incubated overnight at 4 °C with antibodies to ELOVL4 (rabbit polyclonal IgG, 1:1,000) in TTBS. Bound primary antibody was detected by horseradish peroxidase (HRP)-linked donkey anti-rabbit IgG (Invitrogen) secondary antibody at 1:2,000. Protein bands were visualized with a Fujifilm LAS-3000 digital scanner, and quantitated using the Fuji Software. Membranes were then stripped for 30 min and then re-probed with GAPDH (mouse monoclonal IgG), as a loading control, and detected with HRP-chicken anti-mouse IgG (Invitrogen), or mouse anti-actin (1:2,000; A5441, Sigma-Aldrich, St Louis, MO).

## Additional information

**How to cite this article:** Rice, D.S. *et al*. Adiponectin receptor 1 conserves docosahexaenoic acid and promotes photoreceptor cell survival. *Nat. Commun.* 6:6228 doi: 10.1038/ncomms7228 (2015).

## Supplementary Material

Supplementary Information Supplementary Figures 1-5
