# Supplementary material for: Adiponectin receptor 1 conserves docosahexaenoic acid and promotes photoreceptor cell survival
Source: Nat Commun. 2015 Mar 4;6:6228. doi: 10.1038/ncomms7228 (PMC4351799; doi:10.1038/ncomms7228)
Supplement: Supplementary Information — Supplementary Figures 1-5 [file ncomms7228-s1.pdf]

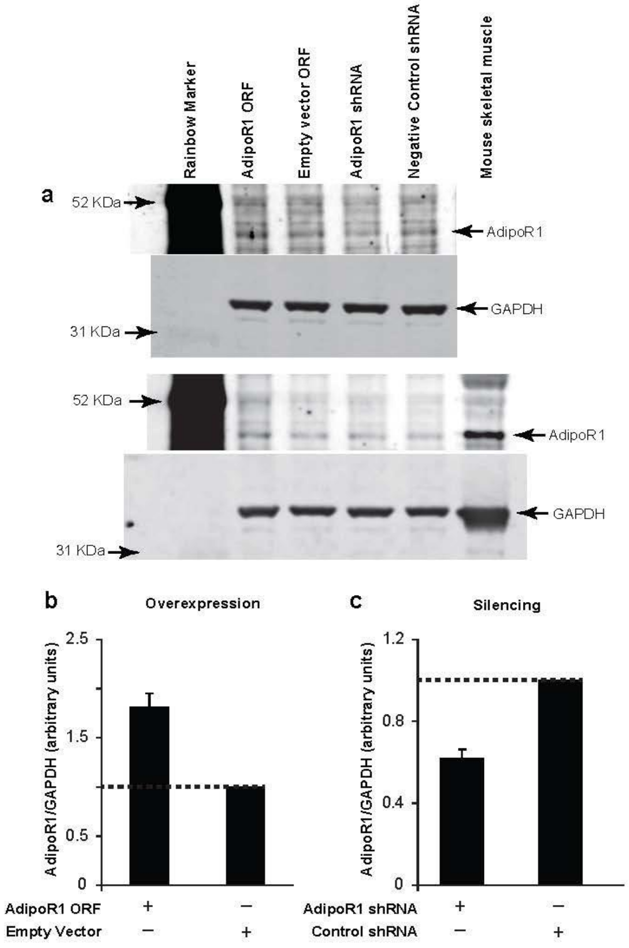

Supplementary Figure 1.

AdipoR1 silencing and overexpression controls. (**a**) Representative blots (upper and lower panels) showing the AdipoR1 protein content relative to GAPDH in two independent experiments. ARPE-19 cells were transfected with a vector containing the human AdipoR1 empty vector, AdipoR1 shRNA, and a non-specific shRNA (negative control). Mouse skeletal muscle lysate was used as a positive control. The first lane displays the marker showing bands at 52 KDa and 31 KDa. (**b, c**) Densitometric measurement of western blots from three independent experiments showing an increase of 81% in AdipoR1 protein content in ORF expressing cells (**b**) and decrease of 38.1% in AdipoR1 silenced cells (**c**). Responses are expressed relative to controls. AdipoR1 was standardized using GAPDH. shRNA, short hairpin RNA; ORF, open reading frame.

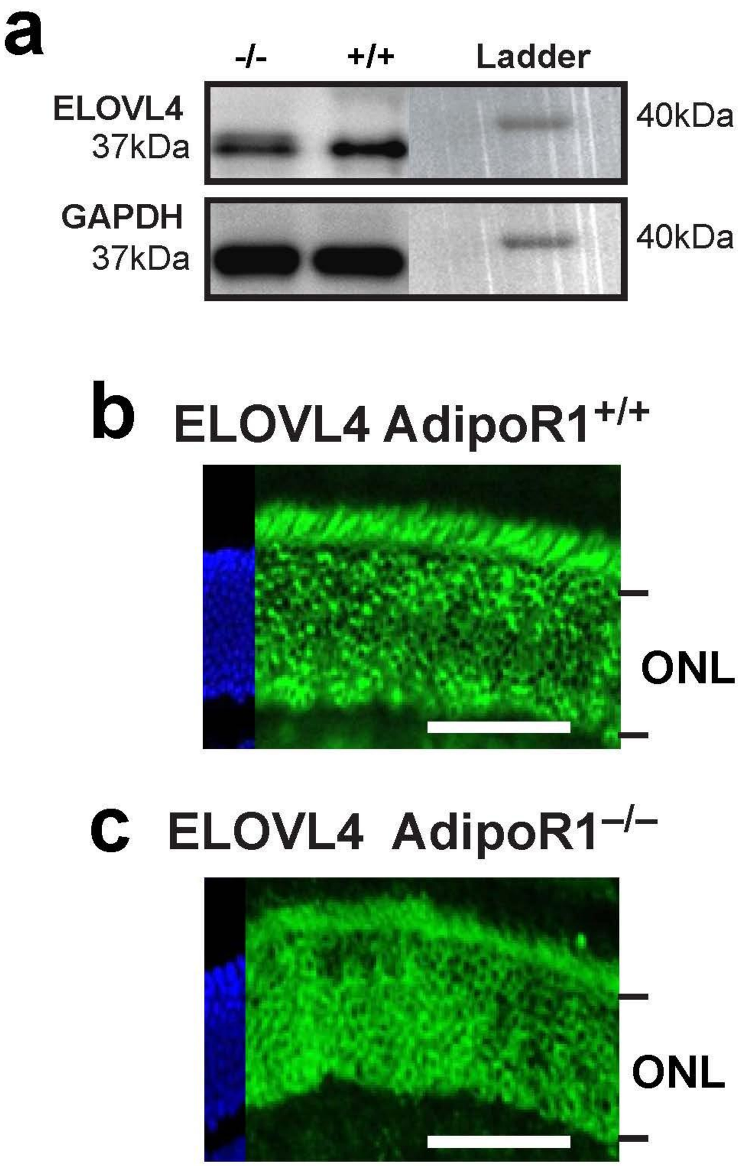

Supplementary Figure 2.

ELOVL4 is not down-regulated in AdipoR1$^{-/-}$ retinas. (a) Western blot demonstrating no significant difference in ELOVL4 concentration between AdipoR1$^{+/+}$ and $^{-/-}$ retinas. **(b)** Immunohistolocalization of ELOVL4 in an AdipoR1$^{+/+}$ retina. Anti-ELOVL4 label (green) appears within the ONL and inner segments of photoreceptors. **(c)** Immunohistolocalization of ELOVL4 in an AdipoR1$^{-/-}$ retina. Anti-ELOVL4 label (green) also appears within the ONL and inner segments of photoreceptors. ONL, outer nuclear layer. Nuclei appear blue (DAPI labeling). 23-day-old mice were used for both western blot analysis and immunolocalization. ONL, outer nuclear layer. Magnification bar, 50 µm.

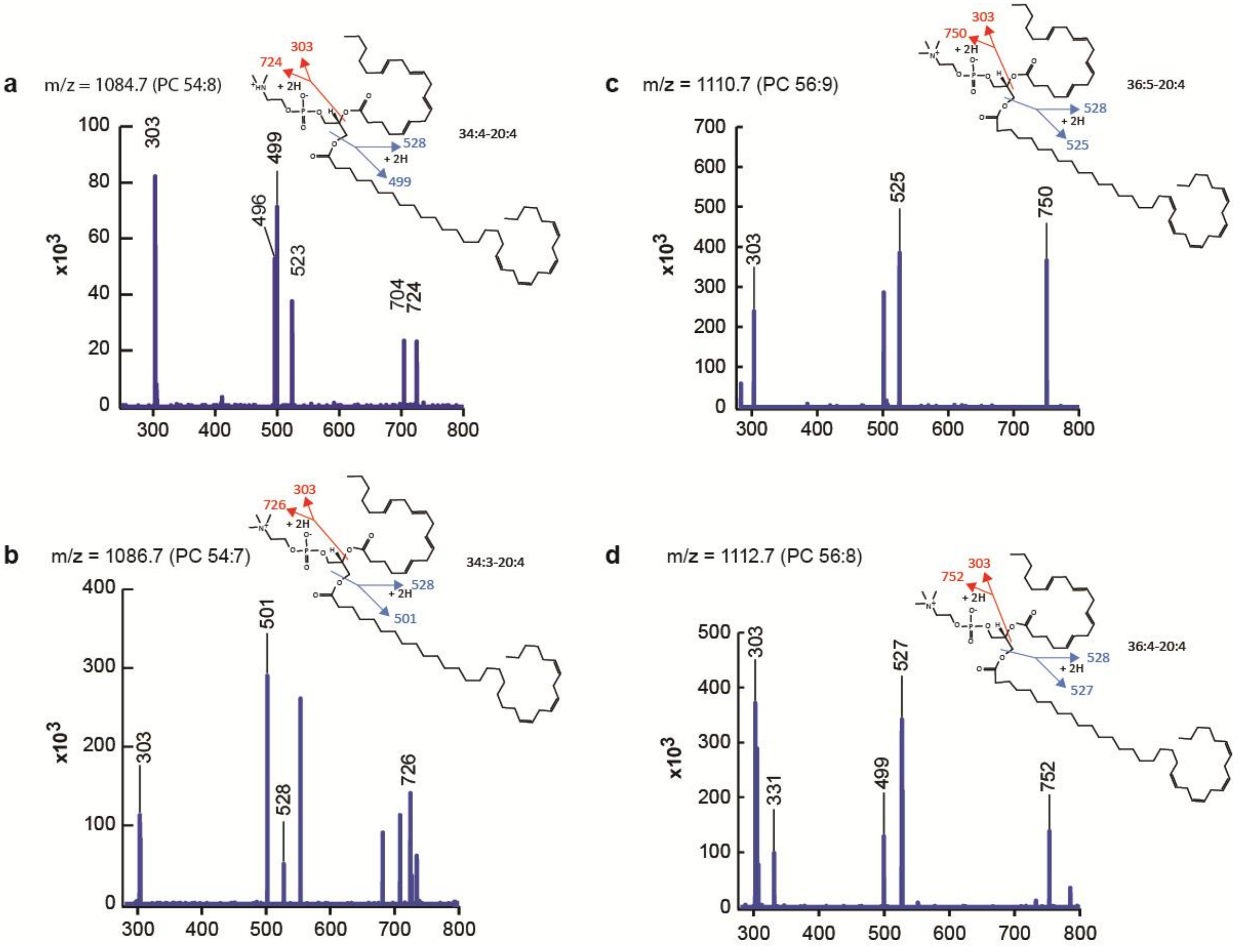

Supplementary Figure 3.
Full fragmentation spectra and molecular structures of $^{-/-}$ VLC-PUFAs. (a) Spectrum of PC 54:8 (m/z = 1084.7 = M(1025.8) + Hac (60) – H) shows the fatty acid composition as FA20:4 and FA34:4. m/z of 724 is produced from (M-(FA20:4-H)+2H, m/z 499 from FA34:4 and m/z 303 from FA20:4. **(b)** Spectrum of PC 54:7 (m/z = 1086.7 = M(1027.8) + Hac (60) – H) shows the fatty acid composition as FA20:4 and FA34:3. m/z of 726 is produced from (M-(FA20:4-H)+2H, m/z 501 from FA34:3, m/z 528 from (M-(FA34:3-H)+2H, and m/z 303 from FA20:4. **(c)** Spectrum of PC 56:9 (m/z = 1110.7 = M(1051.7) + Hac (60) – H) shows the fatty acid composition as FA20:4 and FA36:5. m/z of 750 is produced from (M-(FA20:4-H)+2H, m/z 525 from FA36:5, and m/z 303 from FA20:4. **(d)** Spectrum of PC 56:8 (m/z = 1112.7 = M(1053.7) + Hac (60) – H) shows the fatty acid composition as FA20:4 and FA36:4. m/z of 752 is produced from (M-(FA20:4-H)+2H, m/z 527 from FA36:4, and m/z 303 from FA20:4. m/z of 331 can be from FA22:4, and m/z 499 from 36:5. **(a)** PC54:8 is composed of FA20:4 (arachidonic acid) and FA34:4. **(b)** PC 54:7 is composed of FA20:4 (arachidonic acid) and FA34:3. **(c)** PC 56:9 is composed of FA20:4 (arachidonic acid) and FA36:5. **(d)** PC 56:8 is composed of FA20:4 (arachidonic acid) and FA36:4, or FA22:4 and FA34:4. The molecular structures of these four phospholipids accompany each spectrum.

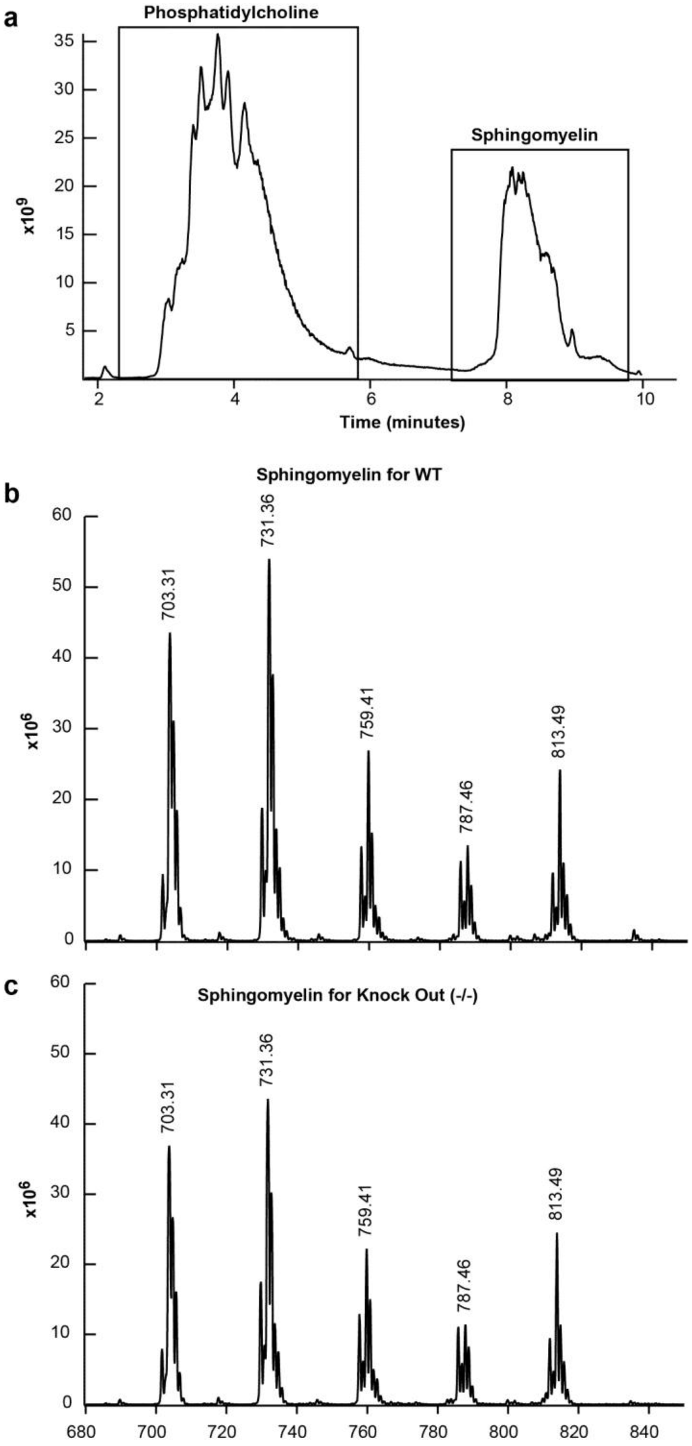

Supplementary Figure 4.
Sphingomyelin levels in AdipoR1$^{+/+}$ and $^{-/-}$ retinas does not change. (a) Chromatogram of retention times for phosphatidylcholine and sphingomyelin, illustrating excellent separation of species. **(b, c)** Mass spectra of AdipoR1$^{+/+}$ and $^{-/-}$ retinas, demonstrating that sphingomyelin levels are not affected by the lack of AdipoR1.

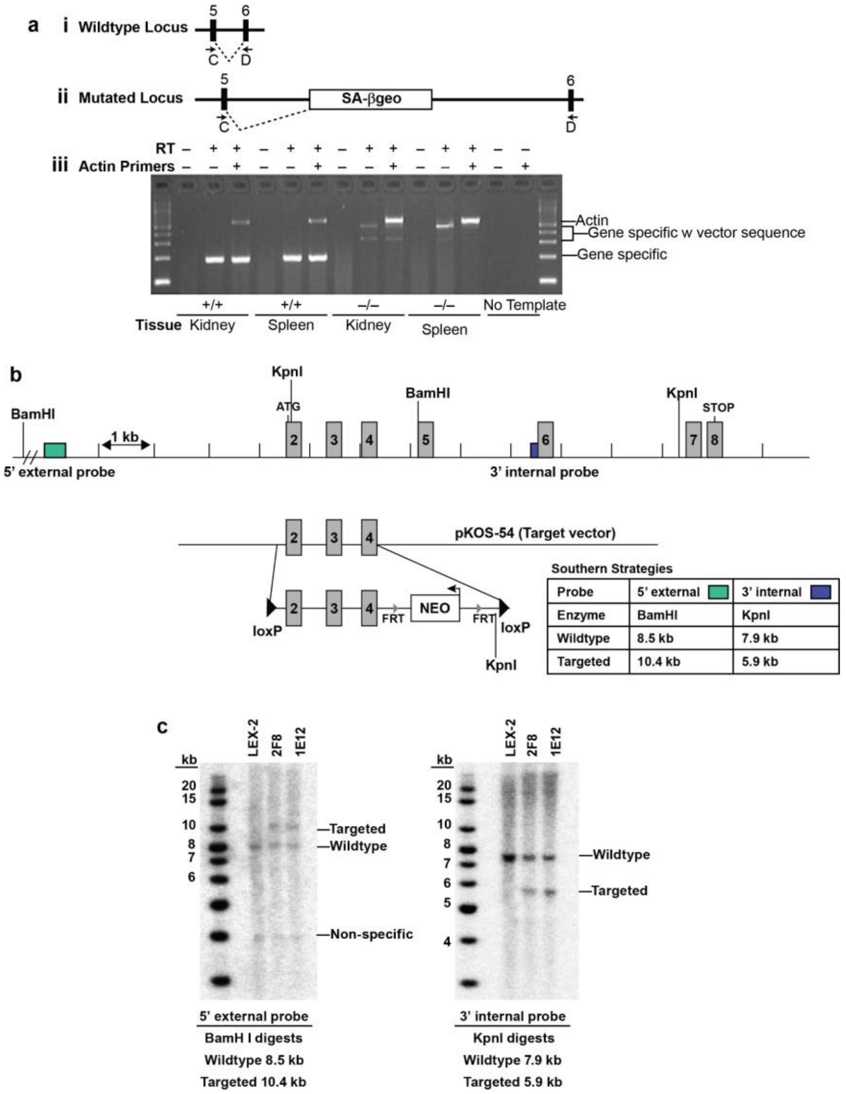

Supplementary Figure 5.

Generation of the AdipoR1$^{-/-}$ mice. (**a**) Design of the gene trap mutation to produce the AdipoR1$^{-/-}$ mice, and the RT-PCR analysis of the AdipoR1$^{+/+}$ and $^{-/-}$ mice to demonstrate the loss of the transcript in the AdipoR1$^{-/-}$ animals. (i) Gene trap mutation of the *AdipoR1* gene. SA, splice acceptor sequence; βgeo, beta galactosidase-neomycin resistance fusion gene. The dotted line in the wild-type (AdipoR1$^{+/+}$) locus indicates normal splicing from exon 5 into exon 6, leading to normal transcript expression. The dotted line in the mutated locus indicates splicing from exon 5 into the gene trap vector rather than exon 6, leading to disruption of the normal transcript. (ii) *AdipoR1* RT-PCR. Primers C and D are complementary to *AdipoR1* exons 5 and 6 flanking the insertion site of the gene trapping vector. (iii) RT-PCR, using primers C and D, shows absence of endogenous message in kidney and spleen of AdipoR1$^{-/-}$ animals. RT, reverse transcription.**Targeted disruption of the AdipoR1 gene locus.** (**b**) Targeting strategy used to disrupt the AdipoR1 locus. Homologous recombination (represented by X) between the targeting vector and the AdipoR1 gene results in the replacement of exons 2-4 with the selection cassette. (**c**) Southern hybridization indicating proper gene targeting in the embryonic stem cell clones.
